# Mulberry leaf extract displays antidiabetic activity in *db/db* mice *via* Akt and AMP-activated protein kinase phosphorylation

**DOI:** 10.29219/fnr.v62.1473

**Published:** 2018-08-22

**Authors:** Ui-Jin Bae, Eun-Soo Jung, Su-Jin Jung, Soo-Wan Chae, Byung-Hyun Park

**Affiliations:** 1 Department of Biochemistry, Chonbuk National University Medical School, Jeonju, Jeonbuk, Republic of Korea; 2 Clinical Trial Center for Functional Foods, Chonbuk National University Hospital, Jeonju, Jeonbuk, Republic of Korea; 3 Department of Pharmacology, Chonbuk National University Medical School, Jeonju, Jeonbuk, Republic of Korea

**Keywords:** glucose uptake, muscle, steatosis, insulin, hypertriglyceridemia

## Abstract

**Background:**

Augmenting glucose utilization in skeletal muscle *via* the phosphatidylinositol-3 kinase (PI3 kinase)/protein kinase B (Akt) pathway or the adenosine monophosphate (AMP)-activated protein kinase (AMPK) pathway is necessary to regulate hyperglycemia in patients with type 2 diabetes mellitus.

**Objective:**

We investigated the effect of mulberry leaf extract (MLE) on glucose uptake in skeletal muscle cells and explored its *in vivo* antidiabetic potential.

**Design:**

Male *db/db* mice were treated with either MLE (50 mg/kg, 100 mg/kg, and 250 mg/kg) or metformin (100 mg/kg) for 8 weeks.

**Results:**

MLE treatment stimulated glucose uptake, driven by enhanced translocation of glucose transporter 4 to cell membranes in L6 myotubes. These effects of MLE were synergistic with those of insulin and were abolished in the presence of PI3K inhibitor or AMPK inhibitor. In *db/db* mice, supplementation with MLE decreased fasting blood glucose and insulin levels and enhanced insulin sensitivity, with increases of p-Akt and p-AMPK in skeletal muscle. Moreover, MLE improved blood lipid parameters and attenuated hepatic steatosis in diabetic *db/db* mice.

**Discussion:**

These findings suggest that MLE exerts antidiabetic activity through stimulating glucose disposal in skeletal muscle cells *via* the PI3K/Akt and AMPK pathways.

**Conclusions:**

MLE can potentially improve hyperglycemia and hepatic steatosis in patients with type 2 diabetes.

Type 2 diabetes mellitus (T2DM) is characterized by insulin resistance (IR), hyperglycemia, and hyperinsulinemia. Skeletal muscle accounts for approximately 70–80% of insulin-stimulated glucose uptake in the postprandial state and plays a key role in maintaining glucose homeostasis (1). Two distinct pathways are responsible for glucose transport in skeletal muscle: phosphatidylinositol-3 kinase (PI3 kinase)/protein kinase B (Akt) and AMP-activated protein kinase (AMPK). Insulin facilitates glucose uptake by increasing the translocation of glucose transporter 4 (GLUT4) from an intracellular pool to the plasma membrane through the activation of the PI3K/Akt pathway (2). Skeletal muscle contraction or metformin treatment activates AMPK and stimulates GLUT4 translocation in an insulin-independent manner (3). Impairment of GLUT4 translocation or decreased GLUT4 activity results in systemic IR (4, 5).

Mulberry (*Morus alba* L.), a member of the Moraceae family, has been cultivated worldwide for sericulture from ancient times and has been used in Chinese medicine to prevent and cure T2DM (6). Results from animal studies show that mulberry leaf extract (MLE) reduces postprandial blood glucose levels in rats with high fat diet- and streptozotocin-induced diabetes (7, 8). Similarly, long-term administration of MLE in subjects with impaired glucose tolerance or T2DM produces a dose-dependent decrease in postprandial blood glucose levels (9, 10). A number of studies have revealed that 1-deoxynojirimycin (DNJ), a potent α-glucosidase inhibitor, is the main component responsible for these activities (11, 12). MLE activates the PI3K/Akt pathway and stimulates glucose uptake in rat adipocytes (13, 14). It increases adipogenesis and stimulates adiponectin secretion from murine 3T3-L1 adipocytes: both activities are associated with decreased blood glucose levels (15). Anthocyanins isolated from MLE maintain the PI3K/Akt pathway and suppress hepatic gluconeogenesis in HepG2 cells, a human hepatocellular carcinoma cell line (14). Given that skeletal muscle is a major site of whole-body glucose uptake and utilization, MLE might also act as a direct stimulant of glucose transport in skeletal muscle in addition to its role in adipocytes and hepatocytes. However, the effects of MLE on skeletal muscle are poorly understood. In this study, using *db/db* mice, we carried out an 8-week supplementation study to ascertain the antidiabetic efficacy of MLE. We also explored the underlying mechanism of action of MLE, with a particular focus on the AMPK and PI3K/Akt signaling pathways, in L6 myotubes.

## Materials and methods

### Preparation of MLE

Dried mulberry leaves were obtained from the Buan Agricultural Development & Technology Center (Buan, Korea) in 2016. Dried mulberry leaf extract was prepared with 15 volumes of water at 50°C for 4 h, using a DH-M03 accelerated solvent extractor (DM Engineering Co., Siheung, Korea). The extracts were filtered using filter cartridges (1 μm), concentrated using a vacuum evaporator (DH-M07, Vacuum Engineering Co., Seoul, Korea) at 60°C, and spray dried with 20% dextrin.

### High-performance liquid chromatography analysis of MLE

The components of MLE were analyzed using an Agilent 1260 Infinity HPLC system (Agilent, Santa Clara, CA, USA) with an MG C_18_ column (4.6 mm × 250 mm, 5 μm, Shiseido Co., Tokyo, Japan). The mobile phase was composed of 0.1% acetic acid in water (A) and acetonitrile (B). The gradient program (A:B) was as follows: 70:30 for 0–16 min, 20:80 for 17–27 min, and 70:30 for 28–35 min. The flow rate was 1 mL/min, the injection volume was 10 μl, and the column temperature was maintained at 35°C. Signals were detected at the wavelengths of excitation (254 nm) and emission (322 nm) using a fluorescence detector. The standard (DNJ) for HPLC analysis was obtained from Sigma-Aldrich (St Louis, MO, USA). The DNJ content was determined using a validation method established using dilutions of each standard at concentrations ranging from 1.03 to 32.90 ppb injected into the HPLC system (correlation coefficient 0.999).

### Liquid chromatography mass spectrometry (LC-MS) analysis of MLE

Unbiased metabolomics analysis was performed using an ultra-performance liquid chromatography (UPLC) system (Waters, Milford, CT, USA). Chromatographic separation was carried out using an ACQUITY UPLC HSS T3 column (100 mm × 2.1 mm, 1.8 μm, Waters) with a column temperature of 40°C and a flow rate of 0.5 mL/min, where the mobile phase contained solvent A (0.1% formic acid in distilled water [DW]) and solvent B (0.1% formic acid in acetonitrile). Metabolites were eluted using the following gradient elution conditions: 97% solvent A for 0–5 min, 3–100% linear gradient solvent B for 5–16 min, 100% solvent B for 16–17 min, 100–3% reverse linear gradient solvent B for 17–19 min; and 97% solvent A for 19–25 min. The loading volume of each sample was 5 μL. The metabolites eluted from the column were detected by a high-resolution tandem mass spectrometer SYNAPT G2 Si HDMS QTOF (Waters) in positive and negative ion modes. For positive ion mode, the capillary voltage and the cone voltage were set at 2 kV and 40 V, respectively. For negative ion mode, they were set at 1 kV and 40 V, respectively. Centroid MSE mode was used to collect the mass spectrometry data. The primary scan ranged from 50 to 1,200 Da and the scanning time was 0.2 sec. All the parent ions were fragmented using 20–40 eV. Information for all fragments was collected, and the time was 0.2 sec. In the data acquisition process, the LE signal was gained every 3 sec for real-time quality correction. For accurate mass acquisition, leucine enkephalin at a flow rate of 10 μL/min was used as a lock mass by a lock spray interface to monitor the positive ([M + H]+ = 556.2771) and the negative ([M − H]− = 554.2615) ion modes. Data acquisition and analysis were controlled using the Waters UNIFI V1.71 software. The scan ranges in MS and MS/MS modes were each 50–1,200 m/z.

### Cell culture and differentiation

Rat myoblast cells (L6) were obtained from the American Type Culture Collection (Manassas, VA, USA). Cells were grown in Dulbecco’s modified Eagle’s medium containing 10% fetal bovine serum, 10 units/mL penicillin, and 10 μg/mL streptomycin, at 37°C and air containing 5% CO_2_. L6 myoblasts were seeded at the density of 2 × 10^4^ cells/cm^2^ in 96-well plates one day before the start of the differentiation. The differentiation of myoblasts into myotubes was induced by switching the media to alpha minimum essential medium (α-MEM) supplemented with 2% fetal bovine serum and antibiotics. Cells were allowed to differentiate into myotubes for 5 days. Fresh medium was added on day 2. The myotubes were then cultured in α-MEM containing 0.2% BSA and antibiotics for 12 h and then used for glucose uptake experiments. Cell viability was assessed using the 3-(4,5-dimethylthiazol-2-yl)-2,5-diphenyltetrazolium bromide (MTT) assay.

### Muscle glucose uptake

L6 myotubes were treated with phosphate buffered saline (PBS), 0.1 μM human insulin (Sigma-Aldrich), and MLE in 0.2% BSA and antibiotics at 37°C in a CO_2_ incubator for 4 h. The cells were washed with PBS and starved for 1 h in glucose-free 4-(2-hydroxyethyl)-1-piperazineethanesulfonic acid (HEPES)-buffered saline (20 mM HEPES, 140 mM NaCl, 5 mM KCl, 2.5 mM MgSO_4_, 1 mM CaCl_2_; pH 7.4) while maintaining the same concentrations of test compounds and insulin. Finally, HEPES-buffered saline containing 10 μM 2-deoxy-D-glucose (2DOG, Sigma-Aldrich) and 0.5 μCi/μL [^3^H]2-deoxy-D-glucose (PerkinElmer, Turku, Finland) was added to the myotubes, and the uptake reaction was allowed to proceed for 15 min at room temperature. The uptake reaction was stopped by washing the cells three times with ice-cold PBS. The cells were lysed in 0.5 M NaOH, and the radioactivity in the lysates was counted by liquid scintillation (PerkinElmer).

### Animals and experimental design

All mice were purchased from The Jackson Laboratory (Bar Harbor, ME, USA). The mice were housed in standard laboratory conditions (23±1°C, 40–60% relative humidity, and a 12 h light–dark cycle) in a barrier facility with laminar flow cabinets in the Laboratory Animal Care facilities of Chonbuk National University Hospital. All mice were allowed free access to a normal chow diet and tap water. After 1 week of acclimatization, 7-week-old male *db/db* mice (C57BLK-*lepr^db^/lepr^db^*) and non-diabetic *db/m* mice (C57BLK-*lepr^db^/*+) were randomly divided into six groups (*n* = 7 per group): normal control (*db/m*, non-treated), negative control (*db/db*, PBS), positive control (*db/db*, 100 mg/kg of metformin), and three test groups (*db/db,* MLE). MLE was administered at doses of 50 mg/kg (low dose), 100 mg/kg (middle dose), and 250 mg/kg (high dose) to the three test groups for 8 weeks. All reagents (PBS, metformin, and MLE) were administered by oral gavage once daily. Food consumption, body weight, and fasting blood glucose (FBG) were measured every week. At the end of the 7th week, the mice underwent an oral glucose tolerance test (OGTT) and an insulin tolerance test (ITT). The animals were sacrificed on the 8th week. Blood sample was collected from the truncal vein. After the mice were anesthetized using ketamine and xylazine, liver and skeletal muscle tissues were harvested. This protocol was approved by the Institutional Animal Care and Use Committee of Chonbuk National University Hospital (permit no. cuh-IACUC-2017-5-1), and all work was performed in accordance with the *Guide for the Care and Use of Laboratory Animals* (National Institutes of Health Publication no. 85-23, revised 2011).

### Biochemical assays

Whole blood glucose levels were measured using Accu-Chek Aviva glucose monitors (Roche Diagnostics, Indianapolis, IN, USA), with blood drawn from the tail vein. Serum insulin and adiponectin levels were measured using an enzyme-linked immunosorbent assay (ELISA) kit (Millipore, Bedford, MA, USA), with blood drawn from the truncal vein. To assess IR, the homeostasis model assessment (HOMA) index scores were calculated as follows: HOMA-IR = (fasting insulin concentration × fasting glucose concentration)/22.5. Serum levels of total cholesterol (TC), triglyceride (TG), and high-density lipoprotein cholesterol (HDL-C) were measured using commercially available kits (Asan Pharmaceuticals Co., Ltd., Seoul, Korea). For quantification of liver TG contents, the liver tissue was homogenized and extracted in chloroform, methanol, and DW (2/1/1 ratio). All procedures were performed according to the manufacturer’s instructions.

### Glucose and insulin tolerance tests

For the OGTT, glucose (1 g/kg body weight) was administered by oral gavage after overnight fasting. The ITT was performed with an intraperitoneal injection of insulin (0.75 U/kg body weight) after 6 h of fasting. These two tests were performed at a 3-day interval and had the same sampling times: 0 (baseline), 15, 30, 60, 120, and 180 min postglucose (or postinsulin) challenge. The glucose levels were measured in the blood drawn from the tail vein. Insulin responsiveness was also assessed in the liver and skeletal muscle. Briefly, insulin (0.75 U/kg body weight) was first administered *via* the truncal vein. Tissue samples were then collected from the liver 5 min later and from the skeletal muscle 10 min later. The levels of total and phosphorylated Akt in each sample were then assessed.

### Histological assay

Liver specimens were immediately fixed in 10% formalin, embedded in paraffin, and cut into 5-μm sections. The specimens were stained with hematoxylin and eosin and examined on a light microscope (Eclipse E600 polarizing microscope, Nikon, Tokyo, Japan).

### Western blotting

Protein samples (20 μg) from L6 myotubes, liver or skeletal muscle tissue extracts were resolved by sodium dodecyl sulfate–polyacrylamide gel electrophoresis and then transferred to nitrocellulose membranes (Millipore). The membranes were probed with primary antibodies against AMPK, p-AMPK, Akt, p-Akt (Cell Signaling Technology, Beverly, MA, USA), GLUT4, and β-actin (Santa Cruz Biotechnology, Dallas, TX, USA) overnight at 4°C. After washing, the membranes were incubated with horseradish peroxidase-conjugated anti-IgG secondary antibodies (Zymed, South San Francisco, CA, USA) for 1 h at room temperature. Immunoreactive bands were detected using an LAS-4000 imager (GE Healthcare Life Science, Pittsburgh, PA, USA), and band density was normalized to that of the β-actin loading control.

### Statistical analysis

Data are expressed as the mean±SEM. Statistical comparisons were made using one-way analysis of variance followed by Fisher’s post hoc analysis using GraphPad Prism 5.02 (San Diego, CA, USA). *P*-values < 0.05 were considered statistically significant.

## Results

### Chromatography analysis of MLE

**Fig. 1:**
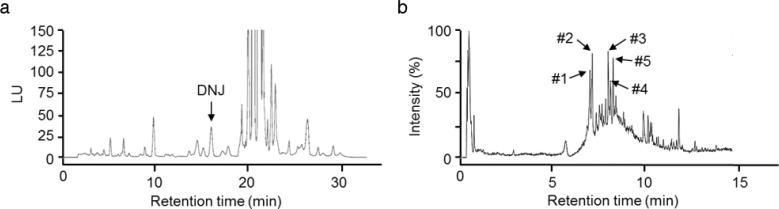
Representative chromatograms of MLE. (a) HPLC analysis of DNJ in MLE. (b) LC-MS analysis of phenolic compounds in MLE. Numbers refer to the main peaks identified: #1, 4-O-caffeoylquinic acid; #2, 1-O-caffeoylquinic acid; #3, kaempferol-3,7-diglucoside; #4, 6-hydroxykaempferol-3-O-glucoside; #5, genistein-7,4’-di-O-β-D-glucoside.

A typical HPLC chromatographic profile of MLE is shown in Fig. 1a. DNJ was adequately resolved from other unknown compounds and could be clearly identified by retention time. The content of DNJ in the extracts was calculated from the relevant peak area by using an external standard method, and quantified as 0.2%. The constituents of the MLE were further analyzed by LC-MS. MLE contained several polyphenols, including caffeoylquinic acid derivatives and flavonoid glycosides (Fig. 1b).

### MLE increases glucose uptake in L6 myotubes

**Fig. 2:**
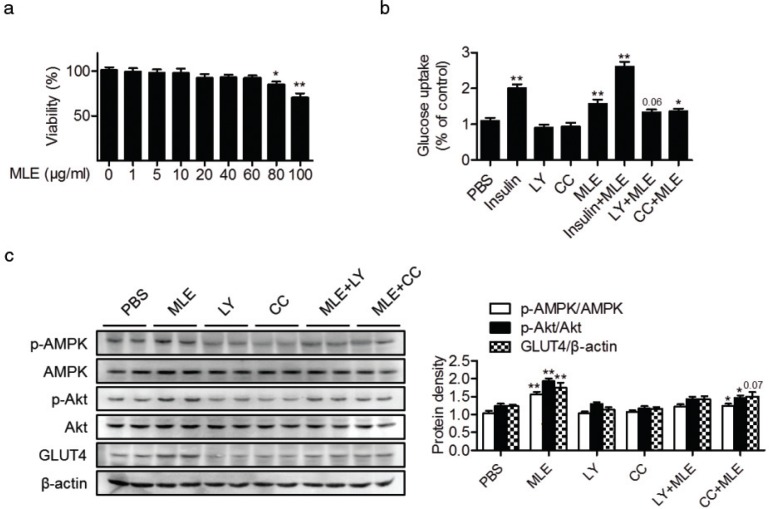
Effects of MLE on glucose uptake in L6 myotubes. (a) L6 myotubes were treated with the indicated concentrations of MLE, and cell viability was determined by MTT assay (*n* = 5). (b) L6 myotubes were treated with MLE (60 μg/mL), 0.1 μM insulin, 25 μM LY294002 (LY), or 10 μM compound C (CC) as indicated. After 6 h, [^3^H]2DOG uptake was measured (*n* = 7/each group). (c) L6 myotubes were treated with MLE (60 μg/mL), 25 μM LY294002, or 10 μM compound C (CC) for 5 min, and protein levels of p-Akt, Akt, p-AMPK, AMPK, and GLUT4 were analyzed by western blotting. The band intensities of each protein were quantitated (*n* = 5). Values are expressed as mean±SEM. MLE, mulberry leaf extract; CC, compound C; LY, LY294002.

To investigate whether MLE has direct effects on muscle cell glucose uptake, L6 myotubes were treated with various concentrations of MLE. The cell viability observed after treatment with ≤60 μg/mL of MLE was similar to that of the control (Fig. 2a). MLE (60 μg/mL) treatment increased [^3^H]-2-deoxy-D-glucose uptake into myotubes (Fig. 2b). Interestingly, glucose uptake was not dependent on the insulin action. To elucidate the mechanism behind this increase in glucose uptake, the levels of p-Akt, p-AMPK, and GLUT4 were measured by western blotting. A significant increase of p-Akt/Akt, p-AMPK/AMPK, and GLUT4 was observed after MLE treatment (Fig. 2c). Using the PI3-K inhibitor LY294002 and the AMPK inhibitor compound C, we further confirmed the activation of the PI3K/Akt and AMPK pathways in the MLE-stimulated glucose uptake (Figs. 2b and 2c).

### MLE decreases blood glucose concentration in db/db mice

**Fig. 3:**
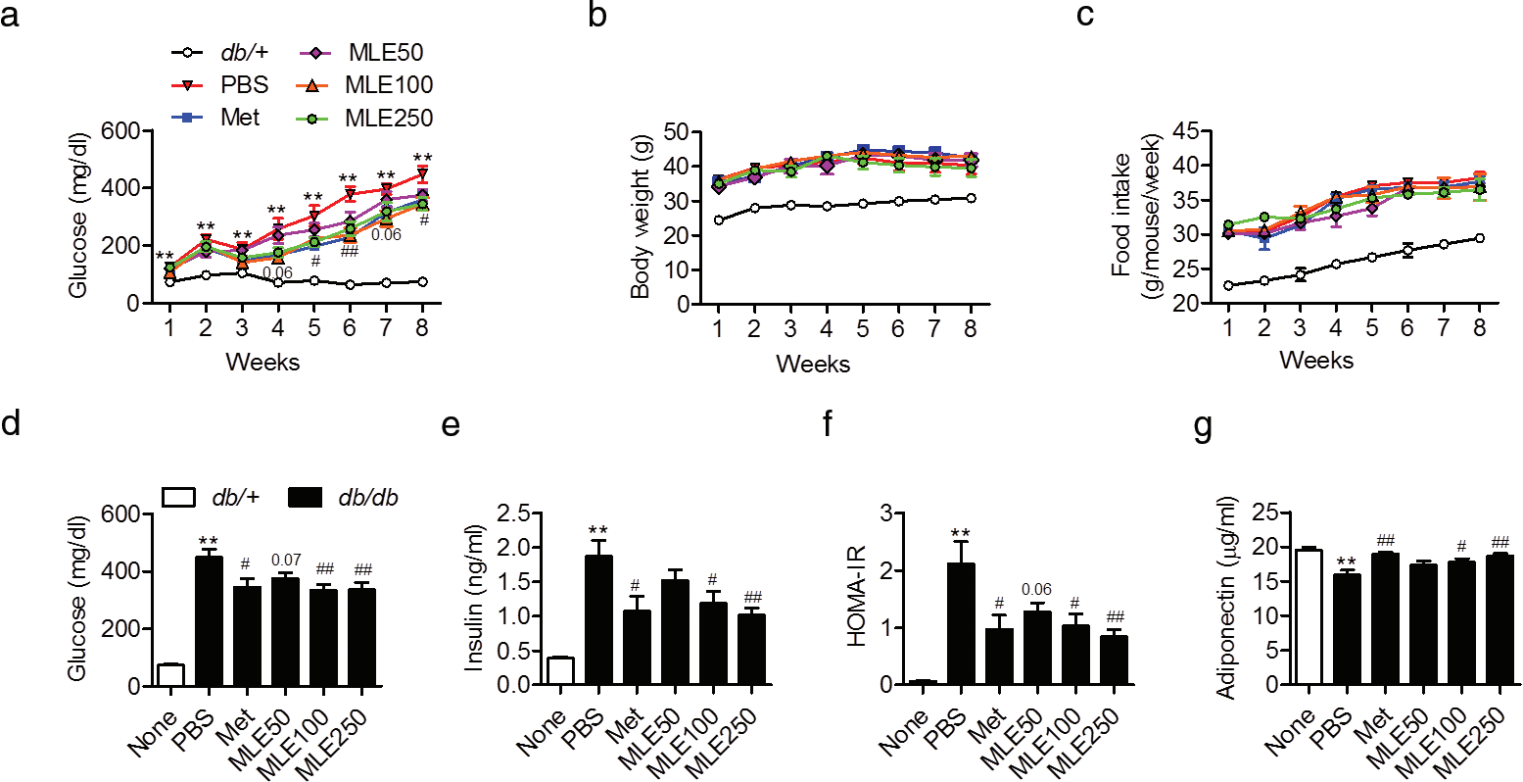
Effects of MLE supplementation on glycemic control in *db/db* mice. Male *db/db* mice received PBS, metformin (100 mg/kg), or MLE (50, 100, and 250 mg/kg) once daily for 8 weeks by oral gavage. (a) Fasting blood glucose, (b) body weight, and (c) food intake changes were recorded at the indicated times. (d–g) At the end of the study, glucose, insulin, and adiponectin concentrations were analyzed and the HOMA-IR scores were calculated. Values are expressed as mean±SEM (*n* = 7/each group). ^**^p < 0.01 versus *db/+*, ^#^*p* < 0.05 and ^##^*p* < 0.01 versus *db/db*+PBS. Met, metformin; MLE50, MLE 50 mg/kg; MLE100, MLE 100 mg/kg; MLE250, MLE 250 mg/kg.

To investigate glucose homeostasis and insulin sensitivity after MLE treatment *in vivo*, we used diabetic *db/db* mice treated with PBS or various concentrations of MLE. Metformin was used as a positive control. All *db/db* mice were confirmed to be diabetic when the experiment began, as indicated by the high mean FBG level (150.10±32.3 mg/dL). After 5 weeks of metformin treatment, the mice exhibited significantly reduced FBG levels compared with the control mice (Fig. 3a). Mice that received 5 weeks of MLE supplementation (250 mg/kg) also exhibited reduced FBG levels; this effect persisted until the end of the study. The glucose-lowering effect of MLE was concentration dependent, and the efficacy of 250 mg/kg MLE supplementation was similar to that of metformin treatment. The average weekly weight gains (Fig. 3b) and food intakes (Fig. 3c) were not different among groups. At the end of the study, the FBG levels, insulin levels, and HOMA-IR scores were significantly decreased in the MLE (100 and 250 mg/kg)-treated *db/db* mice compared with the PBS-treated *db/db* mice (Figs. 3d–3f). Consistent with the changes in FBG levels, MLE supplementation (100 and 250 mg/kg) significantly increased serum adiponectin levels (Fig. 3g).

**Fig. 4:**
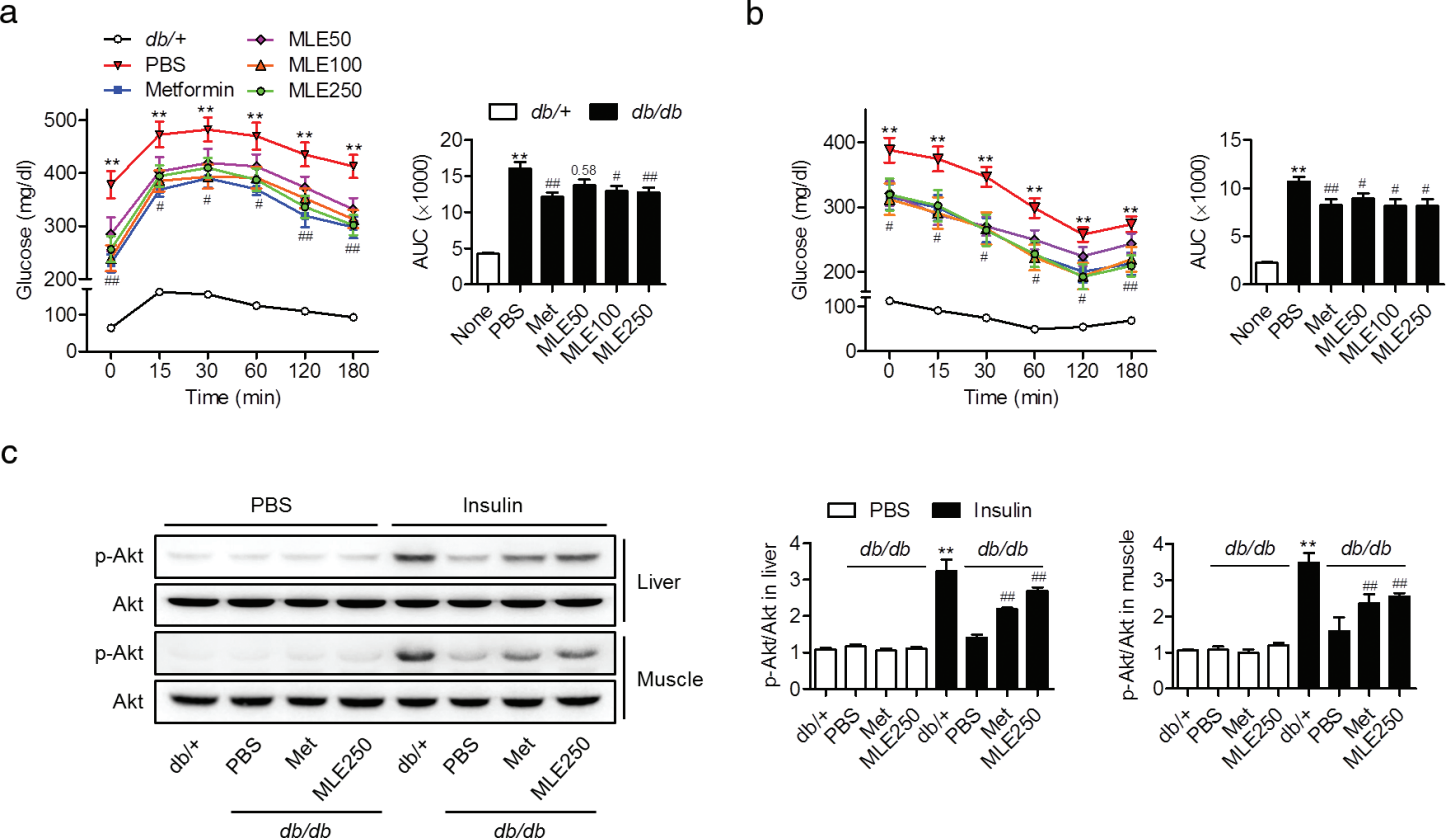
Effects of MLE supplementation on insulin sensitivity in *db/db* mice. At the 7th week, glucose concentrations during oral glucose tolerance tests (a) and insulin tolerance tests (b) were measured. Areas under the curve were compared (*n* = 7/each group). (c) At the end of the study, insulin-stimulated Akt phosphorylation was measured in the liver and skeletal muscle. The band intensities of p-Akt and Akt were quantitated (*n* = 3/each group). Values are expressed as mean±SEM. ^**^p < 0.01 versus *db/+* or *db/+* with PBS, ^#^*p* < 0.05 and ^##^*p* < 0.01 versus *db/db*+PBS or *db/+* with insulin.

Mice with MLE supplementation (100 and 250 mg/kg) also exhibited improvements in post-bolus glucose clearance compared with PBS-treated *db/db* mice at all time points of the OGTT (Fig. 4a). Significant decreases in blood glucose levels during the ITT were also observed in the MLE (50, 100, and 250 mg/kg)-supplemented mice (Fig. 4b). To identify the tissue that contributes to this MLE-mediated increase in insulin sensitivity, the levels of insulin-stimulated Akt phosphorylation were compared in the liver and skeletal muscle tissues. Insulin-injected *db/+* mice showed Akt phosphorylation (Ser473) in the liver and skeletal muscle; however, only weak effects were observed in PBS-treated *db/db* mice (Fig. 4c). In contrast, both MLE (250 mg/kg)- and metformin-treated mice showed significantly increased Akt phosphorylation compared with PBS-treated mice. These results suggest that MLE supplementation improves systemic and peripheral IR in *db/db* mice.

### MLE supplementation ameliorates hepatic steatosis and hypertriglyceridemia in db/db mice

**Fig. 5:**
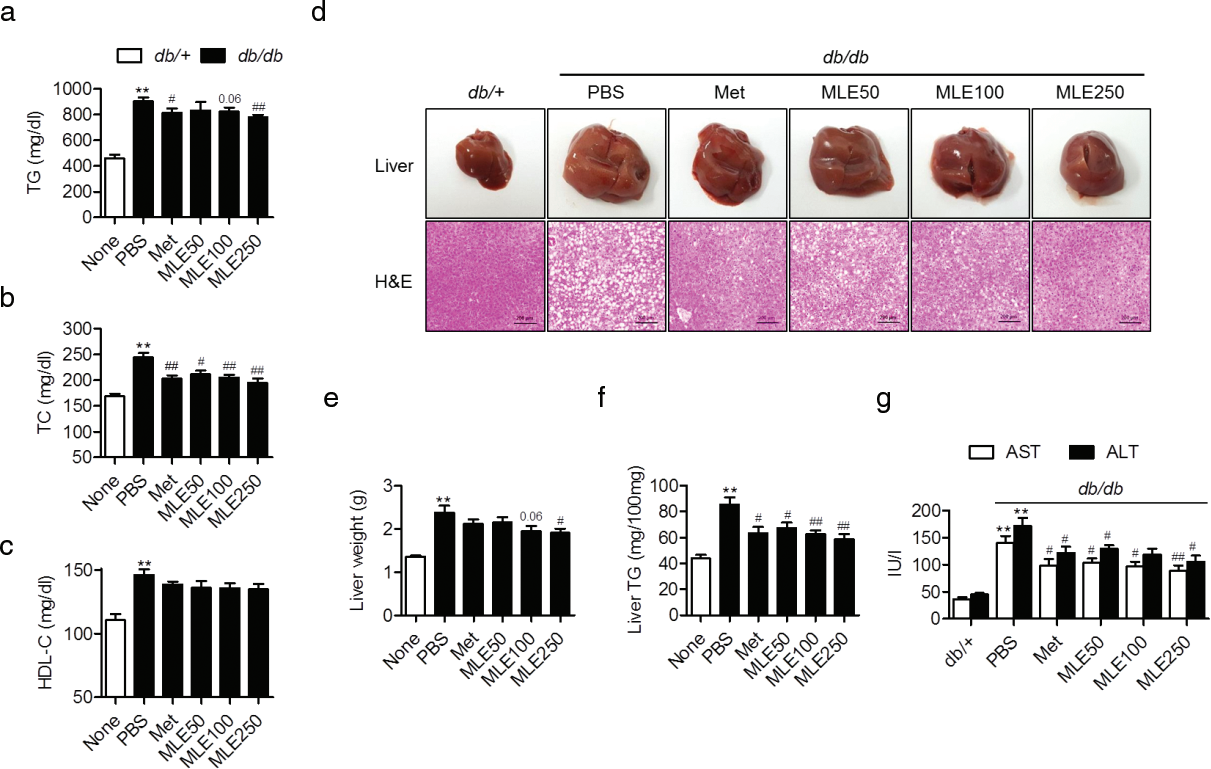
Effects of MLE supplementation on hepatic steatosis and serum lipid profiles in *db/db* mice. (a–c) At the end of the study, serum levels of triglyceride (TG), total cholesterol (TC), and high-density lipoprotein cholesterol (HDL-C) were analyzed. (d) Macroscopic and microscopic images of liver sections. Liver sections were stained with H&E. Bar = 100 μm. (e, f) Liver wet weight and hepatic TG content were determined. (g) Serum levels of AST and ALT were measured as an index of liver injury. Values are expressed as mean±SEM (*n* = 7/each group). ^**^p < 0.01 versus *db/+*, ^#^*p* < 0.05 and ^##^*p* < 0.01 versus *db/db*+PBS.

At the end of the study, supplementation with MLE and supplementation with metformin both resulted in significantly lower serum TG and TC levels in *db/db* mice (Figs. 5a and 5b). However, no significant differences in HDL-C levels were observed with MLE supplementation (Fig. 5c).

Morphological analysis of the livers also indicated that lipid accumulation was most pronounced in the control *db/db* mice. However, supplementation with MLE (250 mg/kg) and metformin both resulted in reduced liver sizes and reduced numbers of lipid droplets compared with *db/db* mice (Fig. 5d). Liver wet weight (Fig. 5e), liver TG content (Fig. 5f), and serum levels of aspartate aminotransferase (AST) and alanine aminotransferase (ALT) (Fig. 5g) were well correlated with the degree of lipid accumulation.

### MLE supplementation activates PI3K/Akt in skeletal muscle

**Fig. 6:**
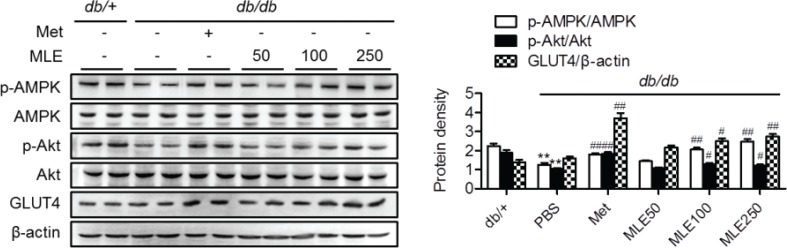
Activation of PI3K/Akt signaling pathways by MLE in muscle. At the end of the study, the total and phosphorylated forms of Akt and AMPK, and GLUT4 were measured in skeletal muscle tissues. The band intensities of each form were quantitated. Values are expressed as mean±SEM (*n* = 7/each group). ^**^p < 0.01 versus *db/+* and ^#^*p* < 0.05, ^##^*p* < 0.01 versus *db/db*+PBS.

Because we observed an increased glucose uptake in L6 myotubes with MLE (Fig. 2), we further verified the improvement of MLE-mediated glucose metabolism in skeletal muscle, the major tissue for glucose disposal. As shown in Fig. 6, phosphorylated forms of Akt and AMPK were significantly decreased in the muscles of PBS-treated *db/db* mice compared with the *db/+* mice. However, supplementation of MLE (100 and 250 mg/kg) significantly increased the phosphorylated forms of these proteins as well as that of GLUT4 protein. These results indicate that MLE likely increases glucose disposal in skeletal muscle by increasing both insulin sensitivity and GLUT4 expression *via* the Akt- and AMPK-dependent pathways.

## Discussion

MLE is rich in the iminosugar DNJ, and therefore much attention has been focused on MLE-dependent suppression of the postprandial elevation of blood glucose levels by delaying glucose absorption in the small intestine. Our LC-MS analysis shows that in addition to DNJ, MLE contains genistein glycoside, kaempferol glycosides, and caffeoylquinic acid derivatives. Previous studies have shown that genistein is responsible for suppressed hepatic gluconeogenesis and increased insulin secretion (16, 17), kaempferol stimulates insulin secretion and exerts insulinotropic effects (18, 19), and caffeoylquinic acid is effective in stimulating glucose transport in muscle cells by activating AMPK and Akt (20). These results suggest that the antidiabetic effects of MLE in *db/db* mice may be mediated by not only DNJ but also a combination of several constituents of MLE.

To determine whether MLE can decrease blood glucose levels in an *in vivo* diabetes model, we treated *db/db* mice with MLE for 8 weeks. Remarkably, MLE supplementation sufficiently lowered FBG levels, and this effect was relatively stable, as with metformin, from 5 weeks after treatment. Because there were decreases in serum insulin levels and HOMA-IR values with MLE supplementation, the insulin sensitization in peripheral tissues is likely the main mechanism by which MLE regulates blood glucose levels. Indeed, our results provide evidence of insulin sensitization with MLE. First, we observed an increase of insulin signaling in the liver and skeletal muscle, as evidenced by the increase of p-Akt/Akt. Second, there was an increase of glucose disposal as shown by an ITT. Third, high levels of adiponectin were detected, which can predict enhanced insulin sensitivity. Finally, GLUT4 expression in skeletal muscle and glucose uptake into L6 myotubes both increased. All of these results indicate that the favorable antidiabetic effects of MLE arise from enhancement of insulin sensitivity in skeletal muscle.

Adipose tissue accounts for a small fraction of glucose disposal after a meal, whereas skeletal muscle is proposed to be the primary site of whole-body insulin-mediated glucose uptake (1). GLUT1 is predominantly responsible for transporting glucose during basal conditions, while GLUT4 becomes the major glucose transporter in skeletal muscle cells in response to insulin (21). Therefore, this study investigated the glucose uptake through GLUT4 in myotubes. Results showed that MLE treatment stimulated glucose uptake independent of insulin action, which was paralleled by increased GLUT4 translocation to the cell surface. The glucose uptake into skeletal muscle is mediated by two pathways: insulin-dependent Akt phosphorylation and contraction-stimulated AMPK activation (2, 3). Studies using the PI3K inhibitor LY294002 and the AMPK inhibitor compound C revealed that both PI3K and AMPK were essential for MLE-enhanced muscular glucose uptake. In agreement with our study, MLE activated AMPK phosphorylation in isolated rat muscle (22); however, in contrast to our results, MLE inhibited Akt signaling in the smooth muscle cell line A7r5 (23). The latter discrepancy is most likely due to using different cell types (skeletal muscle cells in our study vs. smooth muscle cells in their study), as well as visualizing Akt activity at a different MLE concentration (60 μg/mL in our study vs. > 500 μg/mL in their study).

Mice in the MLE treatment group showed decreased serum levels of TG and TC and recovery of fatty liver. Excess glucose enters the liver and is metabolized to acetyl-CoA, which is used for TG synthesis through *de novo* lipogenesis. Insulin and glucose activate SREBP-1c and ChREBP, respectively, and these transcription factors are involved in the induction of lipogenic genes (24). Therefore, downregulation of glucose and insulin levels by MLE may, at least in part, be responsible for the improvement of dyslipidemia and fatty liver in *db/db* mice.

In summary, MLE-supplemented *db/db* mice had not only lowered blood glucose and insulin but also decreased TG and TC levels, and improvements in IR and fatty liver. The mechanism of MLE action involves activation of AMPK and Akt signaling for muscular glucose uptake. Our findings manifest that MLE has a favorable therapeutic potential for the management of type 2 diabetes.
